# Diversity of Serotype, Genotype, and Antibiotic Susceptibility of *Salmonella* Prevalent in Pickled Ready-to-Eat Meat

**DOI:** 10.3389/fmicb.2019.02577

**Published:** 2019-11-12

**Authors:** Jiawei Wang, Huanjing Sheng, Weili Xu, Jinling Huang, Lingyuan Meng, Chenyang Cao, Jie Zeng, Jianghong Meng, Baowei Yang

**Affiliations:** ^1^College of Food Science and Engineering, Northwest A&F University, Yangling, China; ^2^School of Food Science, Henan Institute of Science and Technology, Xinxiang, China; ^3^Department of Nutrition and Food Science, Joint Institute for Food Safety and Applied Nutrition, University of Maryland, College Park, College Park, MD, United States

**Keywords:** *Salmonella* concentration, ready-to-eat food, most probable number, antibiotic susceptibility, pulse-field gel electrophoresis

## Abstract

Pickled ready-to-eat meat (PRTEM) is a meat product that is treated with various seasonings and then cooked. PRTEM is a popular food consumed mostly in China and some Asian countries. Since this food is considered ‘ready to eat’, once it is contaminated by foodborne pathogens such as *Salmonella*, the prospect for significant morbidity, mortality, and immeasurable economic losses can occur. Here we investigated the prevalence and concentration of *Salmonella* in 107 PRTEM samples collected from Shaanxi, China during 2015–2016. Furthermore, we analyzed the serotype, antibiotic susceptibility, and presence of antibiotic resistance genes and amino acid mutations in 219 *Salmonella* isolates, followed by subtyping of 115 representative isolates. The average detection rate of *Salmonella*-positive PRTEM was 58.9%, and the average most probable number (MPN) of *Salmonella* in positive samples was 2.27 logMPN per gram of sample (range: 2.10–2.43). Ten serotypes were identified from the 219 *Salmonella* isolates, with *S.* Thompson (37.9%) and *S*. Indiana (20.5%) being predominant. The remaining serotypes were *S*. Typhi (7.8%), *S*. Typhimurium (7.3%), *S*. Mbandaka (6.9%), *S*. Albany (6.4%), *S*. Blockley (5.5%), *S*. Infantis (4.1%), *S*. Escanaba (3.2%), and *S*. Dusseldorf (0.5%). All isolates were resistant to ceftiofur (100%), while most of them were resistant to ciprofloxacin (99.1%), amoxicillin-clavulanic acid (97.7%), trimethoprim-sulfamethoxazole (96.4%), ampicillin (92.3%), sulfisoxazole (92.2%), tetracyclines (90.4%), and nalidixic acid (90.4%), respectively. A single mutation of Ser83Phe (27.1%) and double mutations of Ser83Phe-Asp87Gly (25.9%) in GyrA were detected in 85 isolates, whereas mutations of Thr57Ser (63.9%) and Ser80Arg (36.1%) in ParC were detected in 122 isolates. *qnr*B, *oqx*AB, *aac(6′)-Ib*, and *qnr*A were present in 50 (22.8%), 48 (21.9%), 26 (11.9%), and 1 (0.5%) isolate(s), respectively. Pulse field gel electrophoresis results revealed that those isolates recovered from the same type of PRTEM or the same sampling place shared identical or similar DNA profiles, antibiotic resistance phenotypes, and even plasmid-mediated quinolone resistance encoding genes. The findings indicate that *Salmonella* is commonly prevalent in PRTEMs at high concentrations in Shaanxi, China. More attention should be paid to the processing and storage of this ready-to-eat food to prevent bacterial contamination and foodborne outbreaks.

## Introduction

*Salmonella* is a genus of Gram-negative bacteria that causes human gastroenteritis, enteric fever, and other fatal diseases. It is recognized as one of the major pathogens for public health and food safety (Liu et al., 2018). According to Majowicz et al. (2010), approximately 94 million people in the world experienced salmonellosis, resulting in 155,000 deaths each year. In China, approximately 70% of bacterial illnesses were due to *Salmonella* in recent years (Chong et al., 2017; Liu et al., 2017; Xing et al., 2018). Currently, *Salmonella* is still an important pathogen associated with foodborne outbreaks and human diseases in the United States and most European countries (Centers for Disease Control [CDC], 2014; European Food Safety Authority (EFSA), 2017).

To date, more than 2610 *Salmonella* serotypes have been identified, among which *Salmonella* Typhimurium, *S.* Indiana, and *S.* Enteritidis are the most common serotypes involved in an increasing number of foodborne outbreaks in some countries (Gong et al., 2017; Li et al., 2017). In animal husbandry, antibiotics have been used as the first choice of treatment to prevent animal diseases. Especially fluoroquinolones, which have been widely used in life-threatening salmonellosis treatment, are often applied in breeding and augmented growth of animals for food (Voss-Rech et al., 2016). Although poultry and poultry products are considered the primary hosts of *Salmonella* (Yang et al., 2011), members of this genus have been recovered from various foods including pork (Sanchez-Maldonado et al., 2016), nuts (Zhang et al., 2017), vegetables (Han et al., 2008), and other retail products (Ed-Dra et al., 2017; Yang et al., 2016; Yang et al., 2010).

Pickled ready-to-eat meat (PRTEM) is a type of spiced food that is usually processed and cooked. Examples include chicken, duck, and pork products with different seasonings, which are extremely popular and mostly consumed in China, especially during summer. Based on data obtained from the China Meat Industry Information Repository, the total amount of ready-to-eat (RTE) meats consumed will approximate 59.7 million tons in 2020 in China, 16.7 million tons of which will be poultry meats (Cheng et al., 2015). Survey results of *Salmonella* prevalence in retail foods across various provinces and cities in China have shown an upward trend in recent years (Wu et al., 2014; Inns et al., 2017; Li et al., 2018). Among various foodstuffs, RTE meats are considered high-risk foods that can and do result in foodborne diseases throughout the world including China (Fang et al., 2012). However, the prevalence and characteristics of *Salmonella* in RTE meats including PRTEM have not been thoroughly investigated.

In this study, we assessed the prevalence and concentration of *Salmonella* in PRTEM in Shaanxi Province, China. Furthermore, we explored the diversity of serotype, genotype, and antibiotic susceptibility of *Salmonella* isolates to better understand the current situation of food safety with respect to RTE meats in China.

## Materials and Methods

### Sample Collection

One hundred and seven PRTEM samples were randomly collected from different supermarkets and retail stalls in the wet markets across Shaanxi Province, China from December 2015 to December 2016. Those PRTEMs are usually made by cooking fresh or frozen livestock and poultry meat including internal organs as the main raw ingredient with the addition of salt, soy sauce and other condiments. For retail, the cooked-bulk meat was often cut into small pieces and mixed with seasonings including salt, vinegar, fresh green onion, chili pepper and some other spices. During the sampling period, each supermarket and retail stall were visited twice, with no more than six samples collected in each market and/or stall. The samples mainly included pickled chicken wing, chicken foot, chicken gizzard, chicken heart, duck neck, duck intestine, duck wing, and duck head (Table 1). After collection, each sample was placed in a separate sterile sampling bag and kept at 4°C before analysis. To avoid bacterial growth in the samples, their transportation time from the sampling places to the laboratory lasted no longer than 2 h. Each sample was aseptically mashed on a clean bench. Then, 25 g of the sample was weighed into a sterile homogeneous bag containing 225 mL of buffered peptone water (BPW; Luqiao Biotech., Beijing, China) and shaken at 100–120 rpm for 5 min. The BPW rinse solution was used for enumeration and isolation of *Salmonella*.

**TABLE 1:** *Salmonella* prevalence and concentration (most-probable-number, MPN) in 107 pickled ready-to-eat meat (PRTEM) samples.

	**Group (number)**	**Percentage (number) of *Salmonella*-positive samples**	**Mean logMPN per gram food**
Chicken (55)	Chicken foot (19)	63.2 (12)	2.14
	Chicken heart (10)	60.0 (6)	2.24
	Chicken wing (15)	46.7 (7)	2.43
	Chicken gizzard (11)	18.2 (2)	2.40
	Subtotal (55)	49.1 (27)^∗^	
Duck (52)	Duck head (8)	85.7 (6)	2.42
	Duck wing (9)	77.8 (7)	2.10
	Duck intestine (16)	75.0 (12)	2.17
	Duck neck (19)	57.9 (11)	2.3
	Subtotal (52)	69.2 (36)	
Marketplace	Wet market (77)	81.8 (63)^##^	2.27
	Supermarket (30)	0 (0)	0

*^∗^Indicates significant differences in the detection rate of Salmonella-positive samples between chicken and duck products (^∗∗^*p* < 0.01, ^∗^*p* < 0.05). ^##^Indicates highly significant differences in the detection rate of Salmonella-positive samples between wet markets and supermarkets (^##^*p* < 0.01).*

### Bacterial Enrichment, Enumeration, and Isolation

The most-probable-number (MPN) technique issued by the Food Safety and Inspection Service (FSIS) of the United States Department of Agriculture (USDA) was used for enumeration of *Salmonella* with some minor modifications (USDA/FSIS, 2014). Briefly, 1. 0-, 0. 1-, and 0.01-mL aliquots of the BPW rinse solution (representing 0.1, 0.01, and 0.001 g of PRTEM) were added to sterile tubes with 9.0, 9.9, and 9.99 mL of BPW, respectively (*n* = 3 each). After the solutions were thoroughly mixed, the tubes were incubated in a shaking bath at 37°C at 100 rpm for 18–24 h.

A portion (0.5 ± 0.05 mL) of each pre-enriched culture was subsequently transferred into 10 mL of tetrathionate broth (TTB, Luqiao Biotech.), whereas 0.1 ± 0.02 mL of the culture was inoculated into 10 mL of modified Rappaport Vassiliadis broth (mRV, Luqiao Biotech.). The inoculated TTB and mRV broths were incubated at 37 ± 0.5°C with shaking at 100 rpm for 18–24 h. A loopful of TTB or mRV culture of each dilution and replicate was streaked onto xylose lysine tergitol 4 agar (XLT4; BD, Biosciences, San Jose, CA, United States) plates and then incubated at 35 ± 2°C for 22–24 h. One to two presumptive *Salmonella* colonies per plate were selected and purified on fresh XLT4 plates.

A single colony with typical *Salmonella* characteristics (e.g., black color, round shape, and smooth surface) was picked and inoculated onto a Luria-Bertani agar (LB; Luqiao Biotech.) plate. The *Salmonella* isolates were confirmed by the agglutination method using *Salmonella* poly A-F antiserum sera (S&A Company, Bangkok, Thailand). The MPN value of *Salmonella* in each sample was determined via the USDA-FSIS MPN table. One isolate from each *Salmonella*-positive TTB and/or mRV tube was selected even if they were derived from the same sample. The *Salmonella* isolates were stored at −80°C in LB broth/glycerol (50/50%, V/V).

### Antibiotic Susceptibility Test

All *Salmonella* isolates were tested for their susceptibility to 15 antibiotics using the agar dilution method (Table 2) developed by the Clinical and Laboratory Standards Institute (Clinical and Laboratory Standards Institute [CLSI], 2014). The category of antibiotics corresponded to that of the National Antimicrobial Resistance Monitoring System (NARMS) managed by the U.S. Food and Drug Administration (USDA) and the Centers for Disease Control and Prevention (CDC). Breakpoint for resistance or susceptibility interpretation to each antibiotic was in accordance with the CLSI standards (Clinical and Laboratory Standards Institute [CLSI], 2014), while the breakpoint for streptomycin was in accordance with that of the NARMS used for susceptibility testing of *Salmonella* and *Escherichia coli* (NARMS, 2011). *E. coli* ATCC 25922 and *Enterococcus faecalis* ATCC 29212 were used as positive control bacteria.

**TABLE 2:** The minimum inhibitory concentration (MIC) ranges and breakpoints of 15 antibiotics used in the study.

**Antibiotic**	**Abbreviation**	**MIC range (μg/mL)**	**MIC interpretive standard (μg/mL)^a^**		
			**Susceptible**	**Intermediate**	**Resistant**
Amikacin	AMK	8–64	≤16	32	≥64
Gentamicin	GEN	2–16	≤4	8	≥16
Kanamycin	KAN	4–64	≤16	32	≥64
Streptomycin^a^	STR	32–64	≤32	N/A	≥64
Amoxicillin-clavulanic acid	AMC	4/2–32/16	≤8/4	16/8	≥32/16
Ampicillin	AMP	4–32	≤8	16	≥32
Ceftiofur	CTX	4–64	≤8	16–32	≥64
Cefoxitin	FOX	4–32	≤8	16	≥32
Ceftriaxone	CRO	2–64	≤8	16–32	≥64
Nalidixic acid	NAL	4–32	≤16	N/A	≥32
Ciprofloxacin	CIP	1–8	≤2	4	≥8
Tetracycline	TCY	2–16	≤4	8	≥16
Chloramphenicol	CHL	4–32	≤8	16	≥32
Sulfisoxazole	SUL	64–512	≤256	N/A	≥512
Trimethoprim/sulfamethoxazole	SXT	0.5/9.5–4/76	≤2/38	N/A	≥4/76

*^*a*^Breakpoints for streptomycin were in accordance with that used for susceptibility testing of *Salmonella* and *Escherichia coli* by the NARMS.*

### Serotyping

The *Salmonella* isolates were serotyped at the Shaanxi Center for Disease Control and Prevention (Xi’an, Shaanxi, China). *Salmonella* O and H antigens were determined via the slide agglutination method using *Salmonella*-specific hyper-immune sera (S&A Company), and the serotype of each isolate was assigned following the Kauffmann–White scheme and the manufacturer’s instructions.

### Detection of Antibiotic Resistance Genes and Amino Acid Mutations

Isolates with nalidixic acid or/and ciprofloxacin resistance were screened by polymerase chain reaction (PCR) for the presence of plasmid-mediated quinolone resistance (PMQR) encoding genes of *qnr* alleles (i.e., *qnr*A, *qnr*B, and *qnr*S), *aac(6′)-Ib*, *qep*A, and *oqx*AB. PCR amplification and DNA sequencing were employed for detection of amino acid substitution of quinolone resistance determining regions (QRDRs) in GyrA and ParC. Primers and annealing temperatures for individual amplification are listed in Table 3.

**TABLE 3:** Polymerase chain reaction primers and annealing temperatures for target genes.

**Target gene**	**Primer**	**Sequence (5′–3′)**	**Annealing temperature (°C)**	**Product size (bp)**	**References**
**Gene amplification and sequencing**					
*gyr*A	*gyr*A-F	ACGTACTAGGCAATGACTGG	56	190	Eaves et al., 2004
	*gyr*A-R	AGAAGTCGCCGTCGATAGAA			
*par*C	*par*C-F	CTATGCGATGTCAGAGCTGG	54	270	Eaves et al., 2004
	*par*C-R	TAACAGCAGCTCGGCGTATT			
**Gene detection**					
*qnr*A	*qnr*A-F	AGAGGATTTCTCACGCCAGG	60	580	Cattoir et al., 2007
	*qnr*A-R	TGCCAGGCACAGATCTTGAC			
*qnr*B	*qnr*B-F	GGMATHGAAATTCGCCACTG	56	264	Cattoir et al., 2007
	*qnr*B-R	TTTGCYGYYCGCCAGTCGAA			
*qnr*S	*qnr*S-F	GCAAGTTCATTGAACAGGGT	57	428	Cattoir et al., 2007
	*qnr*S-R	TCTAAACCGTCGAGTTCGGCG			
*aac(6***′***)- Ib*	*aac(6***′***)- Ib*-F	TTGCGATGCTCTATGAGTGGCTA	55	482	Chi et al., 2006
	*aac(6***′***)- Ib*-R	CTCGAATGCCTGGCGTGTTT			
*qep*A	*qep*A-F	CTGCAGGTACTGCGTCATG	60	403	Chen et al., 2012
	*qep*A-R	CGTGTTGCTGGAGTTCTTC			
*oqx*A	*oqx*A-F	GACAGCGTCGCACAGAATG	62	339	Chen et al., 2012
	*oqx*A-R	GGAGACGAGGTTGGTATGGA			
*oqx*B	*oqx*B-F	CGAAGAAAGACCTCCCTACCC	62	240	Chen et al., 2012
	*oqx*B-R	CGCCGCCAATGAGATACA			

Polymerase chain reaction was conducted in 25 μL reactions containing 0.3 μL of 50 pM each primer, 2.5 μL of 10 × PCR buffer, 2 μL of 2.5 mM of dNTP mix, 0.25 μL of 5 U/μL TaqDNA polymerase (TaKaRa, Dalian, China), 1.5 μL of 25 mM MgCl_2_, 5 μL of template DNA using a MyCircle PCR machine (Bio-Rad, Hercules, CA, United States), and 13.15 μL of double-distilled H_2_O. The PCR conditions were as follows: incubation at 94°C for 10 min, followed by 35 cycles of 94°C for 30 s, an annealing temperature for 30 s, and 72°C for 30 s; and a final extension step at 72°C for 10 min. The PCR products were dyed by red gel and visualized under UV light (Bio-Rad) after electrophoresis.

For GyrA and ParC sequence analysis, the PCR products were stored in a box with dry ice and sent to AuGCT Biotech. (Beijing, China) where DNA sequencing was performed on an Illumina platform (Illumina Inc., San Diego, CA, United States). The obtained sequences were aligned using the online BLAST program^1^. *Salmonella Typhimurium* LT2 was used as a positive control.

### Pulse-Field Gel Electrophoresis

According to the sampling place, sampling time, sample type, and selective *Salmonella* enrichment broth, 115 of 219 isolates were selected for pulse-field gel electrophoresis (PFGE) subtyping with *Xba*I. The PFGE was carried out according to the protocol issued by the CDC for *Salmonella*, *E. coli*, and *Shigella* (Ribot et al., 2006). Briefly, the *Salmonella* isolate was streaked onto LB agar (Luqiao Biotech.) and incubated overnight at 37°C. Appropriate numbers of *Salmonella* cells were first suspended into cell suspension buffer (containing 10 mL of 1 M Tris and 20 mL of 0.5 M EDTA, adjusted to 100 mL with sterile double-distilled H_2_O) and then embedded using Seakem Gold agarose (Lonza, Basel, Switzerland). After cell lysis, the released DNA was digested using 50 U of *Xba*I enzyme (TaKaRa) at 37°C for 1.5–2 h. The digested DNA fragments were separated in 0.5 × Tris–borate-EDTA buffer at 14°C using a ChefMapper electrophoresis system (Bio-Rad) for 20 h. The pulse time for electrophoresis was between 2.16 and 63.8 S. *Salmonella* Braenderup H9812 was used as a standard control.

After electrophoresis, the gel was stained with ethidium bromide, and the DNA bands were illuminated under UV light (Bio-Rad). The results were manually analyzed using BioNumerics v3.0 (Applied-Maths, Kortrijk, Belgium).

### Statistical Analysis

Minitab 18 (Minitab Inc., State College, PA, United States) was used for statistical analysis. Pearson chi-square test was used to determine the differences in the concentration of *Salmonella*, detection rate of *Salmonella*-positive samples, and serotypes in PRTEMs across different places (i.e., wet markets and supermarkets) and different meat types (i.e., pickled chicken and duck meats). The results were compared at the 5% (∂ = 0.05) level to evaluate whether a significant difference was observed. The MPN value of *Salmonella* per gram PRTEM was log-transformed using approximate normality. The relationship between logMPN per gram PRTEM and other variables was assessed using the generalized linear model with the identity link function and adjusted dependency within sample type using generalized estimated equations. Significant differences were considered at *P* < 0.05.

## Results

### Prevalence and Concentration of *Salmonella*

Sixty-three (58.9%) of 107 PRTEMs were positive for *Salmonella*, and the average concentration of *Salmonella* in *Salmonella*-positive samples was 2.27 logMPN per gram PRTEM. The detection rate of *Salmonella*-positive samples significantly (*P* < 0.01) differed between the two types of marketplace. While no *Salmonella*-positive PRTEM was detected in supermarkets, 63 *Salmonella*-positive samples (81.8%, 63/77) were found in retail stalls in the wet markets. The detection rate of *Salmonella*-positive duck products (69.2%, 36/52) was significantly (*P* < 0.05) higher than that of chicken products (49.1%, 27/55). The most common *Salmonella*-positive chicken product was chicken foot (63.2%), whereas for duck products, it was duck head (85.7%; Table 1).

The concentrations of *Salmonella* in *Salmonella-*positive samples ranged from 2.10 to 2.43 logMPN per gram PRTEM. The maximum value of *Salmonella* was detected in chicken wing (2.43 logMPN per gram PRTEM), while the minimum value was detected in duck wing (2.10 logMPN per gram PRTEM). No significant (*P* > 0.05) difference was found in MPN values among the eight types of PRTEM samples that were positive for *Salmonella* (Table 1).

### *Salmonella* Serotypes

During MPN enumeration, a total of 219 *Salmonella*-positive TTB and mRV tubes/cultures were identified. Therefore, 219 isolates (one per positive tube/culture) were recovered in this study and used for subsequent analyses. Among the 219 isolates that were all recovered from samples of retail stalls in the wet markets, 17 (7.8%) were typhoidal *Salmonella* (TS) and 202 (92.2%) were non-typhoidal *Salmonella* (NTS). Among the 17 TS isolates, there were 5 (29.4%) from chicken heart, 4 (23.5%) from chicken gizzard, 4 (23.5%) from duck intestine, 3 (17.7%) from duck neck, and 1 (5.9%) from duck wing. Interestingly, although these TS isolates were derived from different types of PRTEM, they were collected from the same retail stall.

Nine serotypes were identified from the 202 NTS isolates, with *S.* Thompson (83; 41.1%) and *S.* Indiana (44; 21.8%) being the most common serotypes. Other serotypes among the NTS isolates were *S.* Typhimurium (16; 7.9%), *S.* Mbandaka (15; 7.4%), *S*. Albany (14; 6.9%), *S*. Blockley (12; 5.9%), *S*. Infantis (10; 5.0%), *S*. Escanaba (7; 3.5%), and *S.* Dusseldorf (1; 0.5%). Thirty-four isolates derived from chicken wing were all *S.* Thompson, 16 isolates from chicken gizzard were all *S*. Indiana, whereas four isolates from duck head were all *S*. Mbandaka. Additionally, 5, 5, and six serotypes were identified from the isolates that were derived from chicken foot, duck neck, and duck wing, respectively (Table 4).

**TABLE 4:** Distribution of non-typhoidal *Salmonella* serotypes identified in PRTEMs.

	**Number (percentage) of non-typhoidal isolates**								
		**Chicken products**				**Duck products**			
**Serotype**	**Total (*n* = 202)**	**Wing (*n* = 34)**	**Foot (*n* = 31)**	**Gizzard (*n* = 16)**	**Heart (*n* = 9)**	**Neck (*n* = 35)**	**Intestine (*n* = 38)**	**Wing (*n* = 35)**	**Head (*n* = 4)**
Thompson	83 (41.1)	34 (100.0)	9 (29.0)		2 (22.2)	2 (5.7)	25 (65.8)	11 (31.4)	
Indiana	44 (21.8)		3 (9.7)	16 (100.0)		11 (31.4)		14 (40.0)	
Typhimurium	16 (7.9)		4 (12.9)		7 (77.8)	5 (14.3)			
Mbandaka	15 (7.4)					7 (20.0)	3 (7.9)	1 (2.9)	4 (100.0)
Albany	14 (6.9)		4 (12.9)			10 (28.6)			
Blockley	12 (5.9)		11 (35.5)					1 (2.9)	
Infantis	10 (5.0)						10 (26.3)		
Escanaba	7 (3.5)							7 (20.0)	
Dusseldorf	1 (0.5)							1 (2.9)	

**FIGURE 1:**
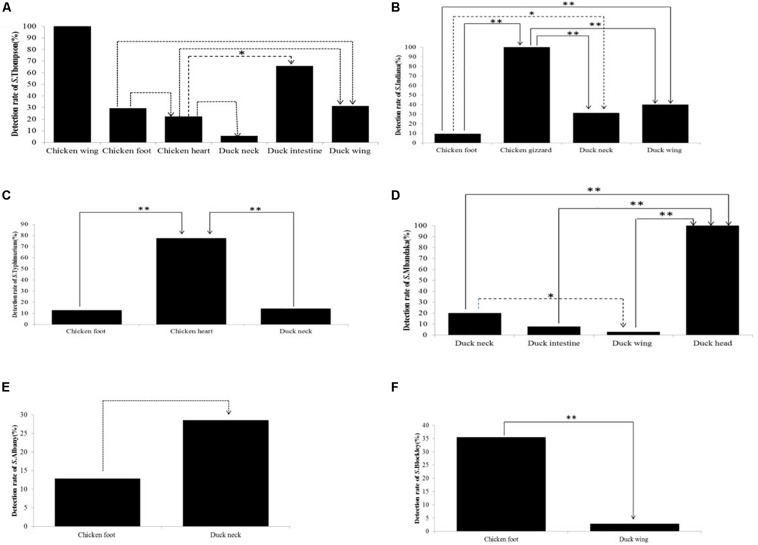
Detection rates of non-typhoidal *Salmonella* strains among the six most common serotypes in different types of *Salmonella-*positive samples from pickled ready-to-eat meats (PRTEMs). **(A)** *S*. Thompson; **(B)** *S*. Indiana **(C)** *S.* Typhimurium; **(D)** *S*. Mandaka; **(E)** *S*. Albany; **(F)** *S*. Blockley. ^∗∗^*P* < 0.01; ^∗^*P* < 0.05.

In addition to chicken wing, *S.* Thompson isolates were also found in chicken foot, chicken heart, duck neck, duck intestine, and duck wing. The detection rate of *Salmonella* Thompson isolates in chicken wing was significantly (*P* < 0.01) higher than those in chicken gizzard and duck head. However, no significant (*P* > 0.05) difference was observed in the detection rate of *S*. Thompson isolates among chicken foot, duck neck, and duck wing (Figure 1A) as well as in the detection rate of *S. Albany* isolates in chicken foot and duck neck (Figure 1E). Similarly, there were significant (*P* < 0.05 or *P* < 0.01) differences in the detection rates of *S*. Indiana (Figure 1B), *S.* Typhimurium (Figure 1C), *S*. Mbandaka (Figure 1D), and *S*. Blockley isolates (Figure 1F) among the *Salmonella* isolates recovered from different types of PRTEMs.

### Antibiotic Susceptibility

All *Salmonella* isolates were resistant to ceftiofur; the detection rate (100.0%) of resistant isolates was significantly (*P* < 0.05) higher than those resistant to the 13 other antibiotics tested for, including amoxicillin-clavulanic acid (97.7%), trimethoprim-sulfamethoxazole (96.4%), ampicillin (92.2%), sulfisoxazole (92.2%), tetracycline (90.4%), and nalidixic acid (90.4%; Table 5). All *Salmonella* isolates were co-resistant to at least five antibiotics, whereas 24 isolates (11.0%) were co-resistant to 5–8 antibiotics, 59 isolates (26.9%) were resistant to 9–12 antibiotics, and 136 isolates (62.1%) were resistant to 13–15 antibiotics tested.

**TABLE 5:** Antibiotic susceptibility of *Salmonella* isolates from PRTEMs.

	**Number (percentage) of resistant isolates**										
**Antibiotic**		**Chicken products**					**Duck products**				
	**Total (*n* = 219)**	**Wing (*n* = 34)**	**Foot (*n* = 31)**	**Gizzard (*n* = 20)**	**Heart (*n* = 14)**	**Subtotal (*n* = 99)**	**Neck (*n* = 38)**	**Intestine (*n* = 42)**	**Wing (*n* = 36)**	**Head (*n* = 4)**	**Subtotal (*n* = 120)**
Ceftiofur	219(100.0)^a^	34 (100.0)	31 (100.0)	20 (100.0)	14 (100.0)	99(100.0)^a^	38 (100.0)	42 (100.0)	36 (100.0)	4(100.0)	120(100.0)^a^
Ciprofloxacin	217(99.1)^ab^	34 (100.0)	31 (100.0)	20 (100.0)	14 (100.0)	99(100.0)^a^	38 (100.0)	42 (100.0)	34 (94.4)	4 (100.0)	118(98.3)^ab^
Amoxicillin-clavulanic acid	214(97.7)^bc^	34 (100.0)	31 (100.0)	20 (100.0)	14 (100.0)	99 (100.0)^a∗^	34 (89.5)	42 (100.0)	36 (100.0)	3 (75.0)	115(95.8)^bc^
Trimethoprim/sulfamethoxazole	211(96.4)^bcd^	34 (100.0)	31 (100.0)	20 (100.0)	11 (78.6)	96 (97.0)^a^	37 (97.4)	40 (95.2)	34 (94.4)	4 (100.0)	115(95.8)^bcd^
Ampicillin	202(92.2)^de^	34 (100.0)	31 (100.0)	20 (100.0)	11 (78.6)	96 (97.0)^ab∗^	29 (76.3)	38 (90.5)	36 (100.0)	3 (75.0)	106(88.3)^e^
Sulfisoxazole	202(92.2)^def^	34 (100.0)	20 (64.5)	20 (100.0)	14 (100.0)	88(88.9)^c^	32 (84.2)	42 (100.0)	36 (100.0)	4 (100.0)	114(95.0)^bcdef^
Tetracycline	198(90.4)^efg^	34 (100.0)	31 (100.0)	20 (100.0)	14 (100.0)	99 (100.0)^abd∗∗^	30 (79.0)	42 (100.0)	26 (72.2)	1 (25.0)	99(82.5)^eg^
Nalidixic acid	198(90.4)^efgh^	34 (100.0)	31 (100.0)	16 (80.0)	8 (57.2)	89(89.9)^ce^	35 (92.1)	37 (88.1)	33 (91.7)	4 (100.0)	109(90.8)^cdefg^
Chloramphenicol	196(89.5)^efgh^	34 (100.0)	31 (100.0)	20 (100.0)	13 (92.86)	98 (99.0)^abd∗∗^	29 (76.3)	41 (97.6)	24 (66.7)	4 (100.0)	98(81.7)^egh^
Ceftriaxone	165(75.3)^i^	34 (100.0)	19 (61.3)	16 (80.0)	9 (64.3)	78(78.8)^cf^	23 (60.5)	28 (66.7)	34 (94.4)	2 (50.0)	87(72.5)^ghi^
Kanamycin	163(74.4)^ij^	34 (100.0)	28 (90.3)	15 (75.0)	9 (64.3)	86 (86.9)^cefg∗∗^	23 (60.5)	28 (66.7)	25 (69.4)	1 (25.0)	77(64.2)^ij^
Gentamicin	147(67.1)^ij^	34 (100.0)	19 (61.3)	15 (75.0)	9 (64.3)	77 (77.8)^fg∗∗^	20 (52.6)	25 (59.5)	25 (69.4)		70(58.3)^jk^
Amikacin	127(58.0)^k^	34 (100.0)	14 (45.2)	5 (25.0)	7 (50.0)	60(60.6)^h^	15 (39.5)	27 (64.3)	25 (69.4)		67(55.8)^jkl^
Cefoxitin	117(53.4)^k^	34 (100.0)	16 (51.6)		8 (57.1)	58(58.6)^h^	10 (26.3)	25 (59.5)	22 (61.1)	2 (50.0)	59(49.2)^kl^
Streptomycin	72(32.9)^l^	5 (14.7)	16 (51.6)	16 (80.0)	2 (14.3)	39(39.4)^i^	15 (39.5)	4 (9.5)	14 (38.9)		33(27.5)^m^

*The same superscript letter in a column indicates no significant difference (*p* > 0.05) in the detection rate of isolates resistant to different antibiotics. ^∗^Indicates significant difference in the detection rate of antibiotic resistance between chicken-borne and duck-borne isolates, while ^∗∗^ indicates highly significant difference (^∗∗^*p* < 0.01, ^∗^*p* < 0.05).*

Among pickled chicken-borne strains, the rates of isolates resistant to ceftiofur (100.0%), ciprofloxacin (100.0%), amoxicillin-clavulanic acid (100.0%), and trimethoprim-sulfamethoxazole (97.0%) were significantly (*P* < 0.05) higher than rates to other antibiotics except for ampicillin (97.0%), chloramphenicol (99.0%), and tetracycline (100.0%). Among pickled duck-borne strains, no significant difference (*P* > 0.05) was found in the detection rates of isolates resistant to ciprofloxacin (98.3%) and ceftiofur (100.0%). However, they were significantly (*P* > 0.05) higher than the rates of isolates that were resistant to the 13 other antibiotics tested for (Table 5).

The detection rates of chicken-borne isolates resistant to amoxicillin-clavulanic acid (100.0%) and ampicillin (97.0%) were significantly (*P* < 0.05) higher than those corresponding resistant isolates derived from pickled duck. Additionally, the detection rates of chicken-borne isolates resistant to tetracycline (100.0%), chloramphenicol (99.0%), kanamycin (86.9%), and gentamycin (77.8%) were significantly higher (*P* < 0.01) than those corresponding resistant isolates derived from pickled duck. Interestingly, all isolates recovered from chicken wing were resistant to 14 antibiotics tested for, except streptomycin. There was a similar situation among isolates recovered from pickled gizzard and duck head (Table 5).

Almost all *S.* Thompson, *S.* Indiana, *S.* Typhi, *S.* Typhimurium, *S.* Mbandaka, *S.* Albany, and *S.* Blockley isolates were resistant to ceftiofur, ciprofloxacin, amoxicillin-clavulanic acid, trimethoprim-sulfamethoxazole, ampicillin, sulfisoxazole (except 1 *S.* Blockley isolate), tetracycline, nalidixic acid (except 2 *S.* Typhi isolates), and chloramphenicol. Among the seven most prevalent serotypes, *S*. Typhi isolates were relatively susceptible to the antibiotics tested for, except for some resistance to ceftiofur, ciprofloxacin, amoxicillin-clavulanic acid, trimethoprim-sulfamethoxazole, sulfisoxazole, tetracycline, and chloramphenicol. These isolates were totally sensitive to kanamycin, gentamicin, amikacin, cefoxitin, and streptomycin. A similar phenomenon was noted for *S*. Blockley and *S*. Mbandaka isolates (Table 6).

**TABLE 6:** Antibiotic susceptibility of *Salmonella* isolates among the seven most common serotypes.

	**Number (percentage) of resistant isolates**						
**Antibiotic**	**Thompson (*n* = 83)**	**Indiana (*n* = 44)**	**Typhi (*n* = 17)**	**Typhimurium (*n* = 16)**	**Mbandaka (*n* = 15)**	**Albany (*n* = 14)**	**Blockley (*n* = 12)**
Ceftiofur	83 (100.0)	44 (100.0)	17 (100.0)	16 (100.0)	15 (100.0)	14 (100.0)	12 (100.0)
Ciprofloxacin	81 (97.6)	44 (100.0)	17 (100.0)	16 (100.0)	15 (100.0)	14 (100.0)	12 (100.0)
Amoxicillin-clavulanic acid	83 (100.0)	42 (97.7)	15 (88.2)	16 (100.0)	14 (93.3)	14 (100.0)	12 (100.0)
Trimethoprim/sulfamethoxazole	81 (97.6)	44 (100.0)	11 (64.7)	16 (100.0)	15 (100.0)	14 (100.0)	12 (100.0)
Ampicillin	81 (97.6)	42 (97.7)	7 (41.2)	16 (100.0)	12 (80.0)	14 (100.0)	12 (100.0)
Sulfisoxazole	81 (97.6)	43 (97.73)	14 (82.4)	16 (100.0)	15 (100.0)	14 (100.0)	1 (8.3)
Tetracycline	81 (97.6)	44 (100.0)	17 (100.0)	16 (100.0)	5 (33.3)	14 (100.0)	11 (91.7)
Nalidixic acid	77 (92.8)	44 (100.0)	2 (11.8)	16 (100.0)	15 (100.0)	14 (100.0)	12 (100.0)
Chloramphenicol	79 (95.2)	25 (56.8)	17 (100.0)	16 (100.0)	15 (100.0)	14 (100.0)	12 (100.0)
Ceftriaxone	82 (98.8)	41 (93.2)	1 (5.9)	14 (87.5)	9 (60.0)	9 (64.3)	1 (8.3)
Kanamycin	81 (97.6)	38 (86.4)		15 (93.8)	7 (46.7)	11 (78.6)	11 (91.7)
Gentamicin	81 (97.6)	40 (90.9)		15 (93.8)		11 (78.6)	
Amikacin	81 (97.6)	25 (56.8)		11 (68.8)		8 (57.1)	
Cefoxitin	80 (96.4)	7 (15.9)		14 (87.5)	4 (26.7)	9 (64.3)	
Streptomycin	6 (7.2)	43 (97.7)		4 (25.0)	3 (20.0)	3 (21.4)	

### Presence of Antibiotic Resistance Genes and Amino Acid Mutations

A total of 237 amino acid substitutions were detected in 219 isolates resistant to nalidixic acid or/and ciprofloxacin. Among these, 115 GyrA mutations in 85 (38.8%) isolates and 122 ParC mutations in 122 (57.7%) isolates were noted. For the GyrA mutations, the most commonly observed was Ser83Phe (23/85, 27.1%), followed by Asp87Asn (9/85, 10.6%), Ser83Tyr (9/85, 10.6%), Asp87Gly (7/85, 8.2%), Val90Leu (4/85, 4.7%), Ala93Ser (1/85, 1.2%), Ala93Val (1/85, 1.2%), and Ser83Thr (1/85, 1.2%). The most frequently observed double mutations in GyrA were Ser83Phe-Asp87Gly (22/85, 25.9%), followed by Ser83Phe-Asp87Asn (4/85, 4.7%) and Ser83Tyr-Asp87Gly (4/85, 4.7%). Mutations in ParC were Thr57Ser (78/122, 63.9%) and Ser80Arg (44/122, 36.1%), respectively. GyrA and ParC mutations were simultaneously detected in 48 (21.9%) *Salmonella* isolates.

No *qnr*S and *qep*A were detected in any isolates. Nonetheless, *qnr*B, *oqx*AB, *aac(6′)-Ib*, and *qnr*A were detected in 50 (22.8%), 48 (21.9%), 26 (11.9%), and 1 (0.5%) isolates, respectively. Twenty-four *Salmonella* isolates co-carried two of the PMQR genes tested for. Ten *Salmonella* isolates had mutations in both GyrA and ParC, while they simultaneously carried *qnr* or *aac(6′)-Ib* genes. Additionally, 29 isolates were detected as having both amino acid mutations and *oqx*AB genes.

### PFGE Subtyping

**FIGURE 2:**
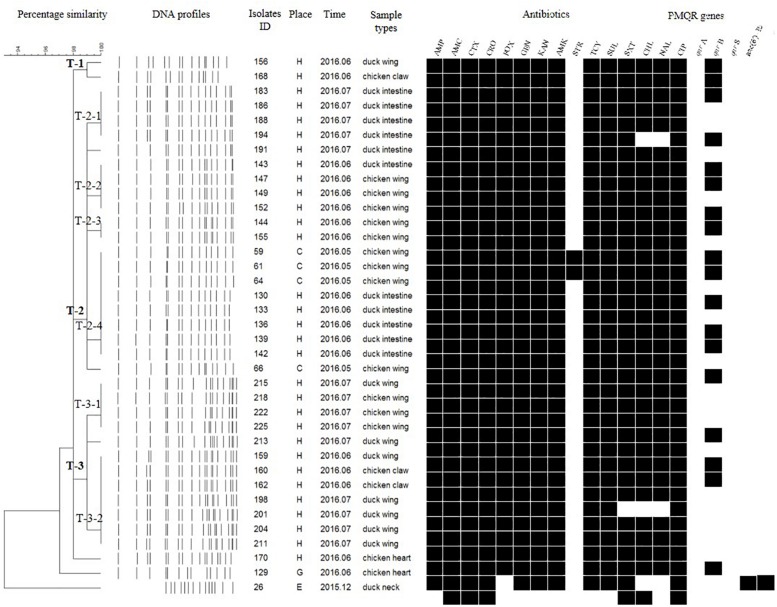
Dendrogram of pulse-field gel electrophoresis (PFGE) profiles for 36 *S.* Thompson isolates, their antibiotic resistance, and plasmid-mediated quinolone resistance (PMQR) genes. Antibiotics: Ampicillin (AMP), Amoxicillin-clavulanic acid (AMC), Ceftiofur (CTX), Ceftriaxone (CRO), Cefoxitin (FOX), Gentamicin (GEN), Kanamycin (KAN), Amikacin (AMK), Streptomycin (STR), Tetracyclines (TCY), Sulfisoxazole (SUL), Trimethoprim/sulfamethoxazole (SXT), Chloramphenicol (CHL), Nalidixic acid (NAL), and Ciprofloxacin (CIP).

**FIGURE 3:**
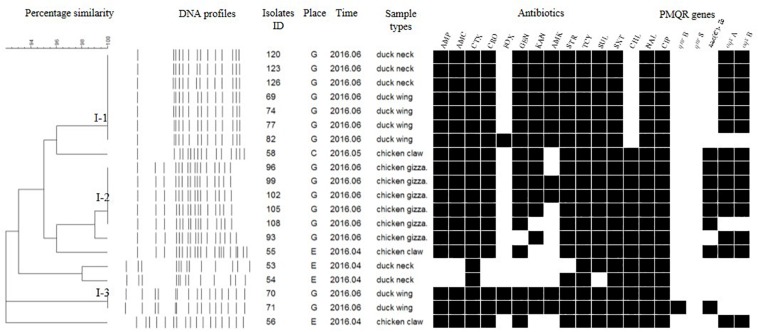
Dendrogram of PFGE profiles for 20 *S.* Indiana isolates, their antibiotic resistance, and PMQR genes. Antibiotics: Ampicillin (AMP), Amoxicillin-clavulanic acid (AMC), Ceftiofur (CTX), Ceftriaxone (CRO), Cefoxitin (FOX), Gentamicin (GEN), Kanamycin (KAN), Amikacin (AMK), Streptomycin (STR), Tetracyclines (TCY), Sulfisoxazole (SUL), Trimethoprim/sulfamethoxazole (SXT), Chloramphenicol (CHL), Nalidixic acid (NAL), and Ciprofloxacin (CIP).

**FIGURE 4:**
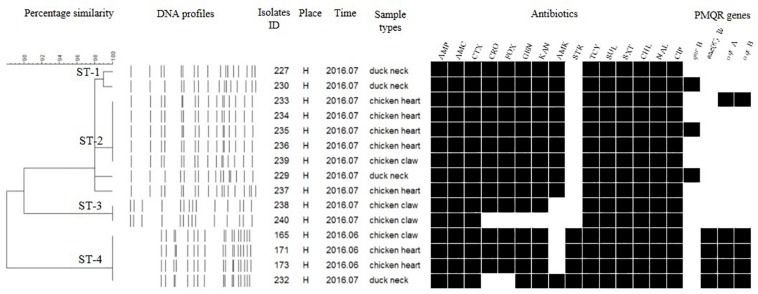
Dendrogram of PFGE profiles for 15 *S.* Typhimurium isolates, their antibiotic resistance, and PMQR genes. Antibiotics: Ampicillin (AMP), Amoxicillin-clavulanic acid (AMC), Ceftiofur (CTX), Ceftriaxone (CRO), Cefoxitin (FOX), Gentamicin (GEN), Kanamycin (KAN), Amikacin (AMK), Streptomycin (STR), Tetracyclines (TCY), Sulfisoxazole (SUL), Trimethoprim/sulfamethoxazole (SXT), Chloramphenicol (CHL), Nalidixic acid (NAL), and Ciprofloxacin (CIP).

**FIGURE 5:**
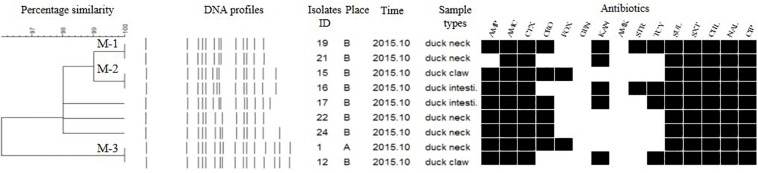
Dendrogram of PFGE profiles for 9 *S.* Mbandaka isolates, their antibiotic resistance, and PMQR genes. Antibitics: Ampicillin (AMP), Amoxicillin-clavulanic acid (AMC), Ceftiofur (CTX), Ceftriaxone (CRO), Cefoxitin (FOX), Gentamicin (GEN), Kanamycin (KAN), Amikacin (AMK), Streptomycin (STR), Tetracyclines (TCY), Sulfisoxazole (SUL), Trimethoprim/sulfamethoxazole (SXT), Chloramphenicol (CHL), Nalidixic acid (NAL), and Ciprofloxacin (CIP).

**FIGURE 6:**
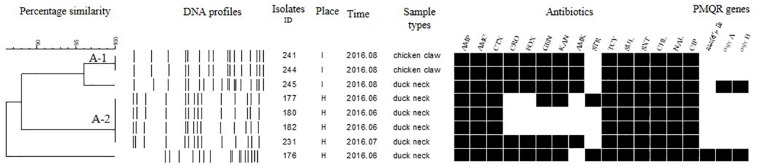
Dendrogram of PFGE profiles for eight *S.* Albany isolates, their antibiotic resistance, and PMQR genes. Antibiotics: Ampicillin (AMP), Amoxicillin-clavulanic acid (AMC), Ceftiofur (CTX), Ceftriaxone (CRO), Cefoxitin (FOX), Gentamicin (GEN), Kanamycin (KAN), Amikacin (AMK), Streptomycin (STR), Tetracyclines (TCY), Sulfisoxazole (SUL), Trimethoprim/sulfamethoxazole (SXT), Chloramphenicol (CHL), Nalidixic acid (NAL), and Ciprofloxacin (CIP).

**FIGURE 7:**
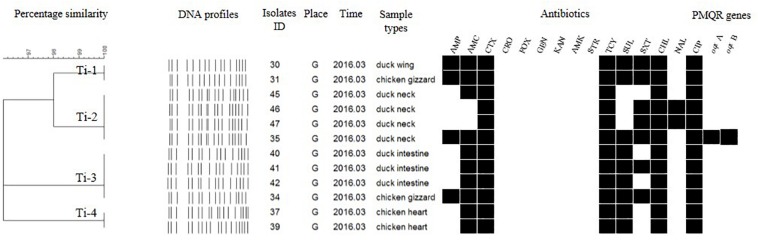
Dendrogram of PFGE profiles for 12 *S.* Typhi isolates, their antibiotic resistance, and PMQR genes. Antibiotics: Ampicillin (AMP), Amoxicillin-clavulanic acid (AMC), Ceftiofur (CTX), Ceftriaxone (CRO), Cefoxitin (FOX), Gentamicin (GEN), Kanamycin (KAN), Amikacin (AMK), Streptomycin (STR), Tetracyclines (TCY), Sulfisoxazole (SUL), Trimethoprim/sulfamethoxazole (SXT), Chloramphenicol (CHL), Nalidixic acid (NAL), and Ciprofloxacin (CIP).

Each isolate produced 13–16 bands with the typing rate of 100%. According to a cut-off value of 90% similarity, 115 isolates were grouped into five clusters (data not shown). PFGE profiles of 103 NTS isolates of the top five serotypes (i.e., *S*. Thompson, *S*. Indiana, *S.* Typhimurium, *S*. Mbandaka, and *S*. Albany) are shown in Figures 2–6, whereas PFGE profiles of the 12 TS isolates are shown in Figure 7.

The *Salmonella* isolates recovered from the same type of PRTEM commonly shared the same DNA profiles, antibiotic resistance phenotype, and even PMQR encoding genes (Figure 2: sub-clusters T-2-1, T-2-3; Figure 3: clusters I-2, I-3; Figure 4: cluster ST-1, ST-3; Figure 6: clusters A-1, A-2). Although some isolates were derived from different types of PRTEMs across various sampling places, they still showed identical or similar DNA profiles, antibiotic resistance profiles, and PMQR genes (Figure 2: cluster T-1, T-2-2, T-2-4, T-3-1, and T-3-2; Figure 3: cluster I-1 and I-2; Figure 4: cluster ST-2 and ST-4; and Figure 5: cluster M-2 and M-3).

For the DNA fingerprints of 12 *S.* Typhi isolates, although some were the same and/or highly similar (98% similarity) in their phylogeny, these isolates could be grouped into four clusters. Isolates derived from the same type of PRTEM were essentially grouped into the same sub-clusters (Figure 7; Ti-2, Ti-3, Ti-4). Moreover, some other strains isolated from different types of PRTEM showed an extremely close phylogenetic relationship (Figure 7, Ti-1, Ti-3).

## Discussion

As a foodborne pathogen, *Salmonella* has resulted in widespread concern and led to socio-economic pressures around the world (Wen et al., 2017). According to data from the USDA, chicken and chicken products are the main vehicles of *Salmonella* and important causes of human *Salmonella* infections; while 37% of chickens have been positive for *Salmonella*, 50–100% of other poultry and eggs have also been shown to carry *Salmonella*^2^ (Voss-Rech et al., 2016). In the current study, we found that all *Salmonella*-positive PRTEM samples were obtained from retail stalls in the wet markets. This result revealed that unsatisfactory hygienic conditions might be the main reason for *Salmonella* contamination in the wet markets. This is especially notable given that no positive samples were detected in supermarkets.

At present, the traditional PRTEM products in China are commonly processed using raw meat with seasonings (i.e., salt, soy sauce, and spices), and water is used as the heating medium. Such PRTEM often contains halogen liquid, with high water content and slightly acidic pH (5.8–6.2) (Zhao et al., 2011). Due to its high water content and adequate nutrition, the PRTEM provides an excellent medium for microbial growth and reproduction (Li, 2015). In most situations, the PRTEM sold in the wet markets is processed by individual peddlers. Thus, the bacterial prevalence in their raw meat, seasonings, and processing environment is largely unknown. Once the raw material is contaminated by pathogens and cross-contamination occurs, the PRTEM will inevitably carry pathogenic bacteria (Zhang et al., 2016).

According to the National Food Safety Standards of China (version GB 29921-2013, GB 4789.4-2016), *Salmonella* should not be detectable in 25 g of cooked or RTE food samples. However, here we found that 63 (58.9%) samples were positive for *Salmonella* in the 107 commercial PRTEM products. This detection rate of *Salmonella*-positive samples is close to the 57.5% of street-vended foods (*n* = 40) in Ethiopia (Mulugeta and Million, 2013), yet it is much higher than the 41.0% of RTE meats (*n* = 79) in Taiwan (Manguiat and Fang, 2013), and the 40.0% of RTE meats (*n* = 20) in Havana, Namibia (Shiningeni et al., 2018). On the other hand, other studies have reported extremely low detection rates of *Salmonella*-positive samples (<4.0%) in RTE meat products (Osaili et al., 2014; Yang et al., 2016; Yang et al., 2018). Together these results indicated that *Salmonella* in RTE meat products throughout the world was more prevalent in specific years tested.

The average concentration of *Salmonella* in the PRTEMs tested in this study was 2.27 logMPN per gram of sample, which is much higher than the level in RTE foods previously reported (Yang et al., 2016). According to the habits of most consumers, PRTEM tends not to be re-processed by heating, seasoning, and/or microwaving before consumption. On the contrary, it is eaten directly as a RTE product. Therefore, the high MPN values reported in this study indicated that *Salmonella*-positive PRTEM foods could pose a serious risk to the health and safety of the consumers. For example, in 2010, a *S.* Typhimurium U323 infection outbreak involving 172 cases occurred in Denmark, which was associated with specific ready-to-eat spreadable pork sausage (Teewurst) (Kuhn et al., 2013). In another national outbreak of *S*. Give in Malta, four restaurants and 26 (72%) human cases were involved, and ready-to-eat antipasti in three of the four restaurants were provided by the same manufacturer (Donachie et al., 2018).

It is very interesting that no *Salmonella*-positive samples were detected in those PRTEMs collected from supermarkets (*n* = 30). However, from retail stalls in the wet markets, the positive rate was as high as 81.8%. Based on our observation and investigation, the following factors might have contributed to the fact that *Salmonella* was only prevalent in the PRTEMs from the wet markets in this survey: (1) PRTEMs sold in the wet markets were typically handled in a family workshop, where the sanitation of the processing environment is more difficult to ensure. Furthermore, the narrow operation space facilitates the chance of cross-contamination between processed PRTEMs and raw meat/poultry products. (2) No sterile vacuum or aseptic packaging was used. During PRTEM processing, long processing times and high temperature treatments can kill almost all bacterial, viral, protozoan, and fungal organisms that are present in the foods, and application of outer and/or inner packaging is an effective procedure in preventing the food from being contaminated. However, no PRTEMs were found to be packaged after processing in these small family workshops. During sale time, such unpackaged PRTEMs are stored at ambient temperatures and completely exposed to the environment, where the chances of further contamination consequently increase. (3) No face masks or gloves were worn by the handlers and sellers during the cutting and weighing of PRTEMs.

Antibiotics are effective chemicals for prevention and treatment of microbial diseases, especially salmonellosis, and thus have been widely used in animal production during the last few decades. However, their usage is also known to promote the occurrence of antibiotic resistant bacteria. According to a latest report, approximately half of the antibiotics in China are commonly used as feed additives in livestock and poultry farming (Chen et al., 2019). Due to the incompleteness of the regulatory system, inadequate interest, and other reasons, manufacturers and farmers have encountered a series of problems such as abuse of antibiotics, which in turn leads to increased bacterial resistance, harm to human health, and environmental damage (Chen et al., 2019). Meanwhile, the types of antifungal drugs currently used for crops are 10 times the types of human and animal drugs (Fisher et al., 2018). With the widespread use of antibiotics for different purposes, many pathogens always exist in a stress environment^3^. Resistance in *Salmonella* thus has shown a continuous upward trend and emerged as a significant public health threat (Voss-Rech et al., 2016; Ed-Dra et al., 2017). Here, we found the 219 *Salmonella* isolates from PRTEM samples were widely resistant to the 15 antibiotics tested in this study. Specifically, the detection rates of nalidixic acid- and ciprofloxacin-resistant isolates were 90.4 and 99.1%, respectively. These values are similar to those rates of resistance previously observed in food-producing animals (Gong et al., 2017; Lai et al., 2014). Although samples in those studies were not RTE foods, the situation and problem of antibiotic-resistant *Salmonella* is both similar and severe.

The rates of nalidixic acid- and ciprofloxacin-resistant *Salmonella* in RTE foods across China (56 and 10%, respectively), to the two antibiotics are considerably higher than those to foods sold in street-vended restaurants in Senegal (0.4 and 1.1%, respectively) (Dione et al., 2009; Yang et al., 2016). This difference may be attributed to the fact that these isolates were recovered from various districts across different time periods, while distinct antibiotics were used across countries and regions. A study also showed that most *Salmonella* isolates in RTE foods were resistant to at least five antibiotics, while some were insusceptible to nine or more antibiotics; 42.0–77.7% of the isolates were multidrug resistant (Xia et al., 2009). In the current study, although some *Salmonella* isolates were susceptible to specific antibiotics, 24 (11.0%) of the total isolates were co-resistant to 5–8 antibiotics, 59 (26.9%) were co-resistant to 9–12 antibiotics, and 136 (62.1%) were co-resistant to 13–15 antibiotics. The implications of these data are both serious and terrifying. In China, resistance of *Salmonella* to antibiotics, especially fluoroquinolones, has been inexorably increasing and expanding on an annual basis (Lai et al., 2014). If the prevalence of *Salmonella* in retail foods including PRTEM is not efficiently prevented and controlled, it is likely that consumers will ingest high concentrations of multidrug-resistant *Salmonella*, which could be fatal.

Mutations in the QRDR are typically associated with nalidixic acid and ciprofloxacin resistance (Gong et al., 2017). Herein, 115 GyrA mutations were detected in 85 (38.8%, 85/219) isolates and 122 ParC mutations in 122 (55.7%, 122/219) isolates. The GyrA mutations mainly occurred as Ser83Phe, Ser83Tyr, Ser83Thr, and Asp87Asn, whereas ParC mutations were Thr57Ser and Ser80Arg. Our result was consistent with those acquired in previous studies (Bai et al., 2015; Bai et al., 2016). In addition to GyrA and ParC mutations, the development of fluoroquinolone resistance was also relative to some penta-peptide repeat proteins encoded by *qnr*, *qep*A, and *oqx*AB genes as well as *aac(6′)-Ib* commonly harbored in the plasmids (Chen et al., 2012; Robicsek et al., 2006). The detection rates of *qnr*B, *oqx*AB, and *aac(6′)-Ib* in our study (22.8, 21.9, and 11.9%, respectively), are in accordance with previously reported data (Chen et al., 2012; Wang et al., 2003). Resistance to fluoroquinolones in *Salmonella* has occurred as a consequence of the environmental and clinical antibiotic usage, amino acid mutations, and emergence of resistance genes (Chen et al., 2019). Despite the relatively high detection rates of antibiotic-resistant isolates in PRTEM samples, point mutations and antibiotic resistance genes were not frequently detected in this study. Because the PRTEMs we collected were pickled and stewed with large amounts of salts, seasonings, and sauces, the stress in external environments might have caused the *Salmonella* isolates to produce multidrug resistance phenotypes.

Ten serotypes were identified in the 219 *Salmonella* isolates recovered in this study, among which *S.* Thompson was the most common serotype (37.9%, 83/219). This differs from previous data showing that *S*. Enteritidis, *S*. Senftenberg, and *S.* Infantis were the predominant serotypes in RTE foods in Thailand (Boonmar et al., 1998), in Henan Province of China (Yu et al., 2014), and in Estonia (Kramarenko et al., 2014). With exception of *S*. Thompson, *S.* Indiana (20.10%; 44/219) was also detected in our study, and this serotype was first reported in the United States in 1955 (James and Carter, 1967), and has not been commonly reported in other countries. However, in China, *S*. Indiana has been more frequently detected annually and predicted to be the second most common serotype in meat products (Gong et al., 2017).

Surprisingly, 17 *S.* Typhi isolates were recovered from PRTEMs at the same sampling place (one of the wet markets) across different sampling times. To our knowledge, isolates of this serotype are typically human-specific pathogens that can cause enteric typhoid fever. *S.* Typhi isolates are commonly recovered from food handlers (Everest et al., 2001; Gebreyesus et al., 2014), water sources (Singh et al., 2015), dairy products (Uzeh et al., 2010), fresh poultry meats (de Freitas et al., 2010), and fresh fruit juice (Diana et al., 2012). No *S*. Typhi has ever been detected from RTE foods such as PRTEM previously. In general, *S.* Typhi cannot inhabit the gastrointestinal tract of livestock; therefore, the contamination of human foods by intestinal contents during domestic animal slaughter is not a means of transmission to humans (Luby, 2014). Although we did not further investigate the *S*. Typhi-positive PRTEM makers for this pathogen, we considered that the following possibilities might exist: (1) the PRTEM makers and/or vendors were *S*. Typhi carriers. Thus, the foods were contaminated during processing by direct contact with the carrier. (2) The water and/or PRTEM processing environment were contaminated by *S*. Typhi. Thus, the processed meats were likely contaminated by *S*. Typhi strains that could survive in PRTEMs in the presence of adequate nutrition for microbial growth.

According to the PFGE fingerprints of *Salmonella* isolates for several of the most commonly detected serotypes, *Salmonella* isolates of each serotype had a very close genetic relationship in PRTEMs during the sampling period, or that they had existed in each of the PRTEM processing environments for a long period. Although these isolates were from different samples across various sampling places and times, they shared the same or very similar DNA profiles, antibiotic resistance phenotypes, and even some antibiotic resistance genes. This phenomenon was also observed for some isolates derived from different food types. Our result indicated that the prevalence of *Salmonella* in PRTEMs could be potential hazards to consumers and would result in *Salmonella* outbreaks in certain time periods.

This study revealed that *Salmonella* was prevalent in commercial PRTEMs at high concentrations. Some of the *Salmonella* isolates recovered from same or different types of PRTEM sheared same or very similar PFGE profiles, antibiotic resistance profiles, QRDR mutations and antibiotic resistance genes. Since PRTEM is a type of RTE food and is generally consumed without re-cooking, the presence of high concentrations of multidrug resistant *Salmonella* is a tremendous public health threat to both food safety and human health.

## Data Availability Statement

All datasets generated for this study are included in the article/supplementary material.

## Author Contributions

JW, JM, and BY proposed the study. HS, WX, and JH collected the samples at the end of the experiment with the assistance of JW. CC and JZ collected the data. JW synthesized the data, retuned and further developed the algorithms, organized, prepared and wrote the first version of the manuscript, and prepared all the figures and tables. All authors contributed to the subsequent versions of the manuscript.

## Conflict of Interest

The authors declare that the research was conducted in the absence of any commercial or financial relationships that could be construed as a potential conflict of interest.

## Footnotes

1 http://www.ncbi.nlm.nih.gov/BLAST/

2 http://www.fsis.usda.gov

3 http://www.sific.com.cn/InsidePage/1000/73/8210.html
